# Impact of maternal obesity on placental transcriptome and morphology associated with fetal growth restriction in mice

**DOI:** 10.1038/s41366-020-0561-3

**Published:** 2020-03-13

**Authors:** Daniela de Barros Mucci, Laura C. Kusinski, Phoebe Wilsmore, Elena Loche, Lucas C. Pantaleão, Thomas J. Ashmore, Heather L. Blackmore, Denise S. Fernandez-Twinn, Maria das Graças T. do Carmo, Susan E. Ozanne

**Affiliations:** 1 Metabolic Research Laboratories and MRC Metabolic Diseases Unit, Wellcome Trust-MRC Institute of Metabolic Science, University of Cambridge, Cambridge, UK; 2 Nutritional Biochemistry Laboratory, Institute of Nutrition Josué de Castro, Federal University of Rio de Janeiro, Rio de Janeiro, Brazil; 3 Nutritional Epidemiology Observatory, Institute of Nutrition Josué de Castro, Federal University of Rio de Janeiro, Rio de Janeiro, Brazil

**Keywords:** Obesity, Animal disease models

## Abstract

**Background:**

In utero exposure to obesity is consistently associated with increased risk of metabolic disease, obesity and cardiovascular dysfunction in later life despite the divergence of birth weight outcomes. The placenta plays a critical role in offspring development and long-term health, as it mediates the crosstalk between the maternal and fetal environments. However, its phenotypic and molecular modifications in the context of maternal obesity associated with fetal growth restriction (FGR) remain poorly understood.

**Methods:**

Using a mouse model of maternal diet-induced obesity, we investigated changes in the placental transcriptome through RNA sequencing (RNA-seq) and Ingenuity Pathway Analysis (IPA) at embryonic day (E) 19. The most differentially expressed genes (FDR < 0.05) were validated by Quantitative real-time PCR (qPCR) in male and female placentae at E19. The expression of these targets and related genes was also determined by qPCR at E13 to examine whether the observed alterations had an earlier onset at mid-gestation. Structural analyses were performed using immunofluorescent staining against Ki67 and CD31 to investigate phenotypic outcomes at both timepoints.

**Results:**

RNA-seq and IPA analyses revealed differential expression of transcripts and pathway interactions related to placental vascular development and tissue morphology in obese placentae at term, including downregulation of *Muc15*, *Cnn1*, and *Acta2*. *Pdgfb*, which is implicated in labyrinthine layer development, was downregulated in obese placentae at E13. This was consistent with the morphological evidence of reduced labyrinth zone (LZ) size, as well as lower fetal weight at both timepoints irrespective of offspring sex.

**Conclusions:**

Maternal obesity results in abnormal placental LZ development and impaired vascularization, which may mediate the observed FGR through reduced transfer of nutrients across the placenta.

## Introduction

The prevalence of obesity has nearly tripled since 1975 [1]. As a consequence, the number of women who are classified as overweight or obese during pregnancy has risen substantially, estimated at 38.9 million in 2014 [2]. This is especially concerning as offspring born to obese mothers are more likely to have poor neonatal outcomes [3] and to develop obesity, insulin resistance, hypertension and dyslipidemia later in life [4]. Interestingly, maternal obesity leads to divergent birth weight outcomes; while it is often shown to increase the risk of macrosomia [5, 6], a higher incidence of low birth weight is also documented [6–8]. Although both are similarly associated with metabolic disease in later life, distinct placental alterations seem to mediate these contrasting offspring phenotypes [9].

Changes in placental function are thought to be pivotal in the development of pregnancy complications [10, 11] and could also be a key link between the maternal and intrauterine *milieu* and long-term health of the offspring [12, 13]. Alterations in placental function and structure in response to obesity and their underlying molecular mechanisms have been explored both in humans and in animal models [9, 14–17]. Yet, even though fetal growth restriction (FGR) is recognized as a placenta-related disorder [18], the impact of maternal obesity on the placental transcriptome in this context remains largely unknown.

It has been shown that placentae from high fat diet-fed obese mouse dams exhibit altered expression of epigenetic machinery genes at term, which could alter the placental epigenome and lead to FGR [19]. High fat diet-induced obesity has also been found to alter the transcriptome of placenta progenitor cells at early stages of development and is associated with later changes in placental function resulting in FGR [17]. In our mouse model of maternal diet-induced obesity, in which dams are fed a hypercaloric Western-like diet, we have shown that maternal hyperinsulinemia is strongly associated with offspring insulin resistance and excess placental lipid deposition and hypoxia [20]. However, a clear understanding of the molecular mechanisms behind these findings is still lacking and warrants further investigation.

It is recognized that the impact of stressors on placental function and offspring health is closely linked to the stage of tissue development, the type of insult and the sex of the conceptus [21]. Thus, the aim of this study was to identify global changes in the placental transcriptome and related pathways in response to maternal obesity near term at embryonic day (E) 19. Furthermore, we investigated whether the significant transcriptional alterations detected in obese placentae were manifested earlier, i.e., in mid-gestation (E13), and if these alterations translated into a structural phenotype in male and female placentae.

## Methods

### Animals and diets

All experimental protocols were approved by the University of Cambridge Animal Welfare and Ethical Review Board and were carried under the Home Office Animals (Scientific Procedures) Act 1986. The model has been described in detail previously [20, 22]. Briefly, female C57BL/6J mice, proven breeders, were randomly assigned either a standard chow RM1 diet [7% simple sugars, 3% fat (wt/wt), 10.74 kJ/g] or an energy-rich highly palatable obesogenic diet [10% simple sugars, 20% animal lard (wt/wt), 28.43 kJ/g] supplemented with sweetened condensed milk [55% simple sugar, 8% fat (wt/wt); Nestle, Croydon, UK], and fortified with mineral and vitamin mix AIN93G. Both diets were fed *ad libitum* and purchased from Special Dietary Services (Witham, UK). Body composition was monitored (TD-NMR, Bruker Minispec) and females were set up to breed if body fat was between the thresholds of 10–12% or 35–40% for Control and Obese dams, respectively. After mating for the second time with RM1 fed males, dams were killed at either E13 or E19 by rising CO_2_ concentration. Fetal and placental weights were recorded. Placentae for molecular analysis were immediately snap frozen on dry ice and stored at −80 °C. For morphological assessment, samples were fixed in 10% formalin for 48 h, stored in 70% ethanol and then embedded in wax.

The sex of the fetuses at E19 was determined by visual inspection of anogenital anatomy. At E13, DNA extracted from tail tips was used for PCR sexing as described by McFarlane et al. [23], using the SX primer pair. Amplicons were loaded on 2% agarose gels and submitted to electrophoresis together with a 1 kb DNA ladder. Bands were visualized with SYBR™ Safe DNA gel stain (Thermo Fisher Scientific, Rochford, UK) under UV-illumination and the genomic sex of each sample was determined according to the number of bands and amplicon size.

### RNA extraction

Placenta aliquots were homogenized in 700-µL Qiazol using TissueRuptor (Qiagen, Manchester, UK). Total RNA was isolated with miRNeasy Mini Kit (Qiagen) according to the manufacturer’s instructions and including the optional step of DNA digestion with RNase-Free DNAse Set (Qiagen). Extracted RNA was quantified by spectrophotometry (Nanodrop^™^ Thermo Fisher Scientific) and stored at −80 °C.

### RNA sequencing and Ingenuity® Pathway Analysis

Total RNA was extracted from E19 male placentae (Control *n* = 2 and Obese *n* = 3), as previously outlined. One microgram of total RNA was depleted of ribosomal RNA and PolyA tails of coding RNAs were captured by treatment with Oligo-dT beads. Complementary DNA (cDNA) libraries were generated after an amplification step, according to the TruSeq Stranded Total RNA Sample Preparation Guide (Illumina, San Diego, CA, USA), and quantified using KAPA Library Quantification Kit. Multiplex single-read sequencing was performed using Illumina HiSeq 2500 (Illumina). Sequence reads were demultiplexed using the CASAVA pipeline (Illumina) and then aligned to the *Mus musculus* genome (GRCm38) using TopHat version 2.0.11. Raw read counts and fragments per kilobase of transcript per million mapped reads (FPKM) were generated using Cufflinks version 2.2.1. A quality check of mapped reads was executed using the R package CummeRbund. Databases were trimmed for exclusion of very low detection or undetectable genes. The resulting data were analyzed using edgeR by calculating the likelihood ratio, and by adjusting *P* values via Benjamini and Hochberg’s method to control the false discovery rate (FDR). Ingenuity Pathway Analysis (IPA) was applied to identify biological pathways related to the genes that were differentially expressed between Control and Obese E19 male placentae. The placenta RNA sequencing (RNA-seq) data have been deposited in GEO database under the accession number GSE140013.

### cDNA synthesis and Quantitative real-time PCR (qPCR)

Total RNA was extracted from male and female placentae of Control and Obese dams at E13 (*n* = 10/group from separate litters) and E19 (*n* = 9/group from separate litters). All samples used in the validations were different to those used in the RNA-seq and therefore represent biological replicates. Sample size was based on previous data sets/power calculations. cDNA was generated from 1-μg RNA using High Capacity cDNA Reverse Transcription Kit (Applied Biosystems, Foster City, CA, USA). qPCR was performed on QuantStudio 7 Flex Real-Time PCR System (Applied Biosystems), using 200 nM specific primers (Sigma-Aldrich, Gillingham, UK), 1× SYBR® Green JumpStart™ *Taq* ReadyMix (Sigma-Aldrich) and cDNA samples at a final dilution of 1:60. For primer sequences see Supplementary Table S1. NormFinder software was used to select the best combination of two out of four reference genes [24]. qPCR results were normalized to the geometric mean of the reference genes *Gapdh* and *Sdha* for E19 placentae, and *Gapdh* and *Pmm1* for E13 placentae, expression of which did not change between groups. Data were expressed in arbitrary units relative to Male Control average (2^−ΔΔCq^).

### Structural analyses

E13 and E19 formalin-fixed placentae from males and females were cut into 5-µm sections. Three serial sections close to the midline of each placenta were selected for staining. Antigen retrieval was performed with pH9 Target Retrieval buffer [S2375 (Dako Agilent, Stockport, UK)] and nonspecific binding sites were blocked with 3% goat serum. Sections were incubated overnight at 4 °C with rabbit polyclonal primary antibodies against Ki67 [1:200; ab15580 (Abcam, Cambridge, UK)] or CD31 [1:500; ab124432 (Abcam)].

Primary antibody binding was visualized by incubation at room temperature for 1 h with a fluorescent-conjugated goat polyclonal anti-rabbit IgG [1:1000; A11008 (Invitrogen, Warrington, UK)]. In negative control slides, placental sections were incubated in 1.5% goat serum in TBS-T instead of primary antibodies (Supplementary Fig. S1). All sections were incubated with 1:2500 DAPI for 10 min at room temperature to stain nuclei. Autofluorescence was quenched by incubation with Vector TrueVIEW Autofluorescence Quenching Kit [SP8400 (Vector Laboratories, Peterborough, UK)].

Placental sections were imaged on an AxioScan Slide Scanner (Zeiss, Cambridge, UK) and blindly analyzed using HALO analysis software (Indica Labs, Corrales, NM, USA). The DAPI channel image was used to define nuclear outlines using the CytoNuclear FL module. For the analysis of Ki67 staining (Supplementary Fig. S2), nuclear outlines were transposed onto the 488-nm channel image and the proportion of nuclei positive for Ki67 across the whole section was recorded. Placental sections stained for the endothelial cell marker CD31 were used to investigate labyrinth zone (LZ) size and structure (Supplementary Fig. S3). The border of the LZ was outlined manually at ×40 magnification (depicted in yellow) and total area was recorded using HALO analysis software after canals were excluded. The boundary with the junctional zone was determined as the interface between the phenotypically distinct spongiotrophoblast of the junctional zone and the fetal capillaries of the LZ. The rest of the boundary was either at the edge of the tissue image or at the interface with the chorionic plate, which is structurally distinct from the LZ, characterized by smaller nuclei and the absence of CD31-positive endothelial cells. Indica Labs’ Tissue Classifier module was used to differentiate between fetal blood vessels (lumen bound by CD31-positive endothelium) and other tissue of the LZ.

### Statistical analyses

Benjamini–Hochberg multiple testing correction [25] was applied to the RNA-seq differential expression data and only genes with FDR < 0.05 were considered significantly different between the two experimental conditions. For qPCR validation of RNA-seq differentially expressed genes, comparisons were made between Control and Obese placentae of the same sex, by Student’s *t* test.

Anthropometric parameters and qPCR of selected targets were analyzed by two-way analysis of variance (ANOVA) followed by Tukey’s multiple comparisons test to estimate the effects of maternal obesity and fetal sex at each time point.

For the morphological analyses, the effects of gestational age (E13 or E19), offspring sex (male or female), and maternal diet (regular chow or obesogenic diet) on placental phenotype were investigated by three-way ANOVA, and backwards stepwise elimination was used to come to a minimal model. Three-way ANOVA of the proportional area of the LZ that was fetal capillaries data revealed no significant effect of gestational age, maternal diet, or fetal sex. However, there was a borderline significant interaction between maternal diet and fetal sex (*P* = 0.055). In order to identify if a maternal diet effect was only present in one sex, these data were separated and sex-specific two-way ANOVAs (gestational age/maternal diet) were performed.

Only one sample from each litter was used for each analysis, except in the case of offspring and placental weights, in which each litter’s average was used as a single data point. Data are presented as mean ± standard error of the mean (SEM), and the threshold for significance was set at *P* < 0.05, unless stated otherwise. Statistical analyses were performed using R (R Core Team 2017) or Prism 6 (GraphPad Prism, La Jolla, CA, USA).

## Results

### Fetal and placental measurements

Fetal and placental weights were reduced in response to obesity and female placentae were smaller than those of males at both stages of gestation. There was no significant difference in the ratio of fetal to placental weight, although there was a trend (*P* = 0.05) toward higher placental efficiency in females at E19 (Table 1).

**Table 1:** Fetal and placental weights at E13 and E19.

	Control		Obese		*P* value	
	Males	Females	Males	Females	Maternal obesity	Sex
E13						
Fetal weight (g)	0.17 ± 0.01	0.16 ± 0.00	0.15 ± 0.01	0.14 ± 0.01	**0.003**	0.187
Placental weight (mg)	94.0 ± 2.2	88.9 ± 3.5	89.7 ± 2.1	78.7 ± 2.9	**0.015**	**0.008**
Fetal:placental ratio	1.84 ± 0.06	1.85 ± 0.06	1.66 ± 0.08	1.76 ± 0.08	0.079	0.452
E19						
Fetal weight (g)	1.23 ± 0.03	1.17 ± 0.03	1.02 ± 0.03	1.03 ± 0.02	**<0.0001**	0.339
Placental weight (mg)	93.8 ± 5.1	81.7 ± 5.3	82.8 ± 1.9	73.1 ± 2.7	**0.039**	**0.021**
Fetal:placental ratio	13.45 ± 0.76	14.77 ± 0.92	12.38 ± 0.37	14.28 ± 0.78	0.331	0.051

Values are mean ± SEM. *P* values < 0.05 indicated in bold show significant effect of maternal obesity and sex differences in the studied parameters according to two-way ANOVA followed by Tukey’s multiple comparisons test, using each litter’s average as a single data (Control Male *n* = 6 and 9, Control Female *n* = 6 and 9, Obese Male *n* = 7 and 7, Obese Female *n* = 7 and 6, respectively, at E13 and E19).

### RNA-seq, Ingenuity® Pathway Analysis and qPCR at term placentae

**Fig. 1:**
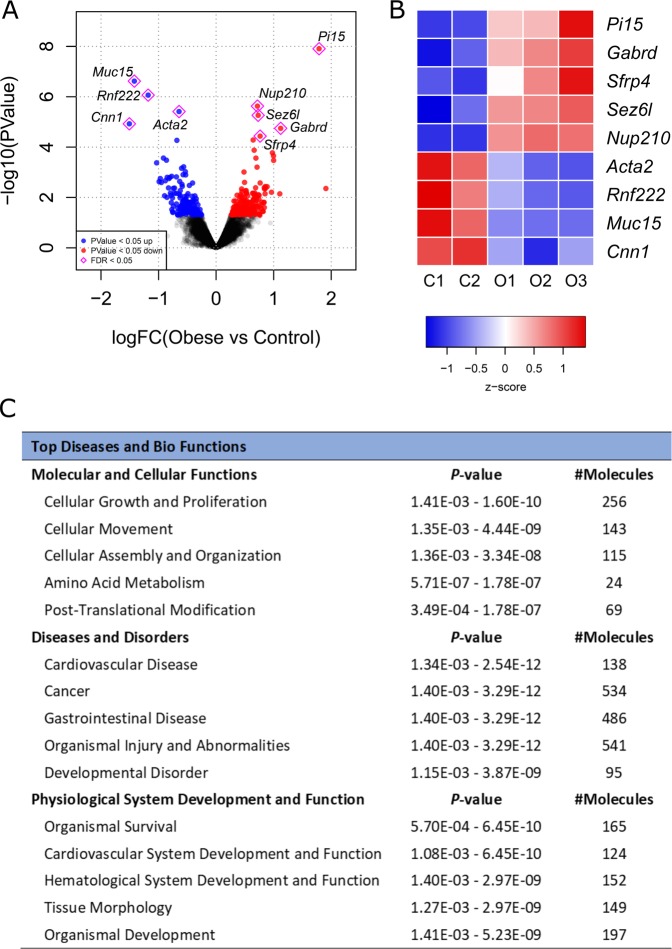
RNA-seq identification of differentially expressed genes between Control (C, *n* = 2) and Obese (O, *n* = 3) male mouse placentae at E19. **a** Volcano plot representing all detected transcripts, distributed according to −log10 *P* value in the *y*-axis and log2 fold change in the *x*-axis, with downregulated genes shifted to the left (*P* < 0.05 in blue) and upregulated genes shifted to the right (*P* < 0.05 in red). Significantly altered genes after correction for multiple testing (FDR < 0.05) are depicted with a pink diamond. **b** Heatmap representation of genes significantly regulated by maternal obesity using a cutoff FDR < 0.05, with scaled *Z*-score color key of normalized counts showing expression levels ranging from blue (lower) to red (higher). Genes are sorted from lowest to highest log2 fold change value**. c** Top diseases and bio functions from Ingenuity® Pathway Analysis (IPA) of the RNA-seq data with a threshold of *P* < 0.05, showing the most significant molecular and cellular functions dysregulated in the placenta by maternal obesity, sorted by *P* value.

The RNA-seq at E19 detected a total of 350 transcripts differentially expressed in placentae of Obese compared to Control males considering a significance threshold of *P* < 0.05 (Fig. 1a, Supplementary Table S2). However, only nine genes remained significantly altered after correction for multiple testing (FDR < 0.05) (Fig. 1a, b). Ingenuity® Pathway Analysis (IPA) was used with a less stringent threshold (*P* < 0.05) to identify global changes in pathways and biological functions promoted by maternal obesity. The most significant diseases and bio functions are shown in Fig. 1c.

**Fig. 2:**
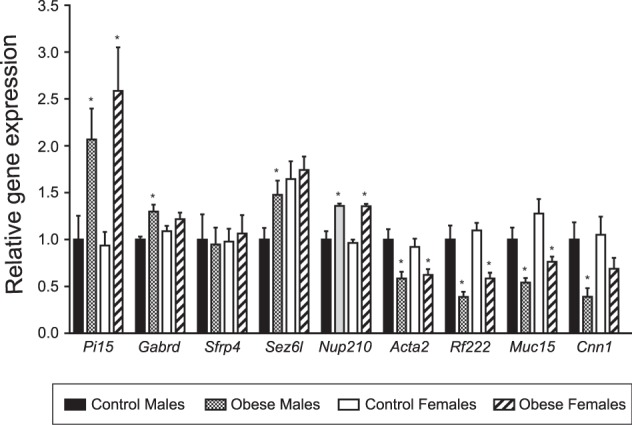
Validation of RNA-seq data by qPCR in E19 male and female placentae. qPCR results were normalized to the reference genes *Gapdh* and *Sdha* and are expressed as mean ± SEM in arbitrary units relative to Male Controls. *P < 0.05, determined by Student’s *t* test comparing qPCR data of same sex Control vs Obese, *n* = 9/group.

Genes identified as significantly changed in response to obesity by RNA-seq (FDR < 0.05) were validated in a larger number of samples, all from independent litters, by qPCR (Fig. 2) and results were confirmed in eight out of the nine genes in E19 male placentae (i.e., *Pi15*, *Gabrd*, *Sez6l*, *Nup210*, *Acta2*, *Rnf222*, *Muc15*, and *Cnn1*). These genes were also examined in female placentae, however, only *Pi15*, *Nup210*, *Acta2*, *Rnf222*, and *Muc15* were significantly modulated by obesity (Fig. 2).

### Placental gene expression at different gestational ages

**Fig. 3:**
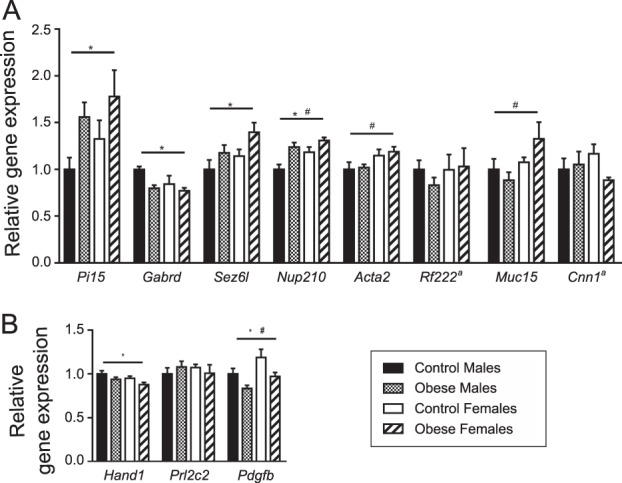
qPCR expression in E13 male and female placentae. **a** Validated RNA-seq genes**. b** *Hand1*, required for trophoblast giant cell (TGC) differentiation; *Prl2c2*, a marker of spiral artery-associated TGC and canal-associated TGC; *Pdgfb*, a growth factor that regulates placental labyrinthine layer development. qPCR data were normalized to the reference genes *Gapdh* and *Pmm1*. Results are shown as mean ± SEM in arbitrary units relative to Male Control average expression. *Denotes maternal obesity effect (*P* < 0.05) and # denotes sex difference (*P* < 0.05), according to two-way ANOVA, *n* = 10/group. ª*Rnf222* and *Cnn1* expression levels were low at E13 placentae, with average Cq values above 31 and 29, respectively.

All nine genes found differentially expressed in obese male placentae at E19 were then investigated in E13 placentae (Fig. 3a). *Pi15*, *Nup210*, and *Sez6l* were upregulated by maternal obesity in both sexes at mid-gestion. *Gabrd* mRNA levels, which were upregulated in Obese male placentae at E19, was downregulated by maternal obesity at E13. *Muc15*, *Nup210*, and *Acta2* expression was higher in females compared to males at E13. *Rnf222* and *Cnn1* were not differentially expressed in either sex at E13, however, their transcript levels were very low compared to E19.

Since genes involved in spiral artery remodeling (*Muc15* and *Cnn1*) and labyrinthine pericytes (*Acta2*) that were found to be dysregulated in obese placentae at E19 were not affected at E13, we additionally measured the expression of candidate genes recognized as relevant for these processes at mid-gestation. Both *Hand1* and *Pdgfb* were downregulated by maternal obesity in both sexes, but no differences were observed in *Prl2c2* (Fig. 3b).

### Immunofluorescent staining of placental morphology

Phenotypic analyses were also conducted by immunofluorescent staining of targets within pathways identified by IPA. The marker Ki67 was used to investigate cellular growth and proliferation. Cellular movement, assembly, and organization were assessed through analyses of LZ size and fetal vasculature structure, using CD31 as a marker of fetal endothelial cells.

**Fig. 4:**
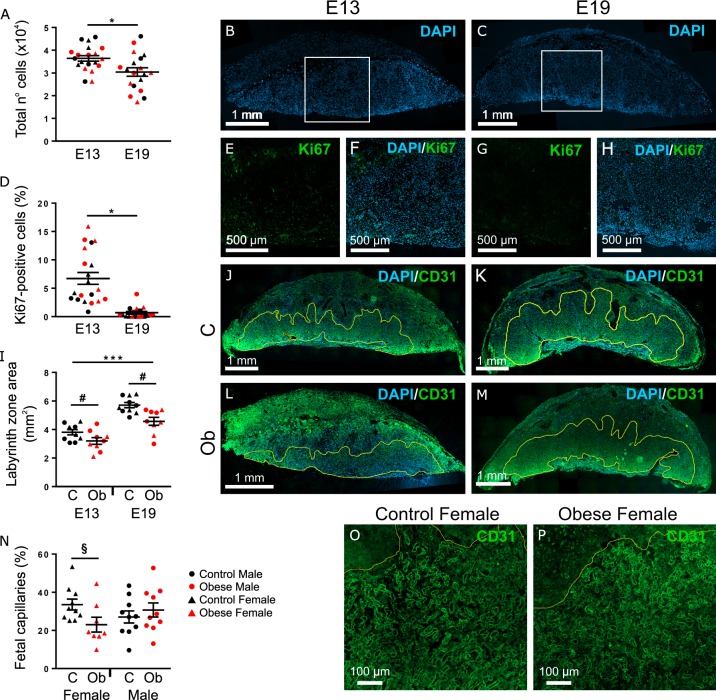
Immunofluorescent staining of targets related to the top three molecular and cellular functions shown in IPA. All analyses were conducted in both male and female placentae of mothers fed either regular chow (C, Control group) or obesogenic diet (Ob, Obese group), at E13 and E19. **a**–**c** The total number of cells in the placenta decreased between E13 (*n* = 20) and E19 (*n* = 19). **d**–**h** The proportion of Ki67-positive cells across the whole placenta decreased between E13 (*n* = 20) and E19 (*n* = 19). **i**–**m** The size of the labyrinth zone increased between E13 and E19, and was reduced in response to maternal obesity (C *n* = 10, Ob *n* = 9, at each time point). **n**–**p** The proportion of fetal capillaries within the labyrinth zone was decreased by maternal obesogenic diet in females (C *n* = 10, Ob *n* = 9). **a**, **d**, **i**, **n** Results are shown as mean ± SEM. Gestational age differences are denoted by *P < 0.05, **P < 0.001, or ***P < 0.0001, and maternal obesity effect is indicated by ^#^*P* < 0.001, according to three-way ANOVA. §Denotes maternal obesity effect (*P* < 0.05), determined by two-way ANOVA analysis of E13 and E19 female placentae only.

E19 placentae had fewer cells (*P* < 0.05, Fig. 4a–c) and a lower proportion of proliferating cells across the whole placenta (*P* < 0.01, Fig. 4d–h) compared to E13. These parameters were not affected by offspring sex or maternal diet.

The size of the LZ was significantly reduced in placentae from obese dams (*P* < 0.01, Fig, 4i–m). There was a significant increase in LZ size from mid-gestation to term (*P* < 0.01, Fig. 4i). Labyrinthine vascular organization was analyzed by the proportion of the total LZ area that was fetal blood vessels. A reduced model considering males and females separately by two-way ANOVA (gestational age/maternal condition) detected a reduction in area bound by fetal capillaries within the LZ in female placentae of obese dams (*P* < 0.05, Fig. 4n–p). Representative images of male placentae are shown in Supplementary Fig. S4.

## Discussion

The RNA-seq analysis revealed a total of 350 transcripts differentially expressed in Obese male placentae at term. Among the top downregulated transcripts, *Muc15, Cnn1* and *Acta2* were of particular significance as these genes are required for appropriate development of placental vasculature and related to key pathways identified by IPA such as cellular movement, assembly and organization. Previous studies have shown that *Muc15* suppresses the migration/invasion of trophoblast like-cells in vitro, a process implicated in blood vessel remodeling in the maternal–fetal interface [26]. *Cnn1* is largely expressed by smooth muscle cells [27] which line the uterine blood vessels and are lost in the normal remodeling of maternal spiral arteries during placental development [28]. *Acta2* is a marker of pericytes which surround fetal endothelial cells during blood vessel development in the mouse LZ [29].

These data together suggest that exposure to maternal obesity affects the remodeling of maternal spiral arteries and the development of fetal blood vessels within the LZ, both of which are crucial for adequate nutrient and oxygen transfer across the placenta [28] and possibly linked to the uteroplacental hemodynamic alterations present in preeclampsia and intrauterine growth restriction [30–32]. Obese women are two to three times more likely to develop preeclampsia [33] and hypertensive obstetric complications are generally associated with small-for-gestational age neonates [34]. Here, we see significant growth restriction in the fetus which may result from poor uteroplacental perfusion in addition to placental hypoxia previously suggested in this model [20].

Furthermore, the RNA-seq analysis identified several genes that have not been functionally described in placental tissue thus far, but are conserved in humans and rodents. *Pi15* encodes a peptidase inhibitor that may regulate extracellular matrix modifications [35] and has been implicated in vascular defects in rat aorta [36], though its role in placental vascularization is unknown. *Gabrd* gene encodes the delta subunit of gamma-aminobutyric acid type A receptor (GABA_A_). GABA_A_ activation impacts stromal cell proliferation and apoptosis during decidualization [37] and increased expression of its pi subunit (*GABRP*) has been detected in preeclamptic placentae [38]. NUP210 is a major component of the nuclear pore complex and is required for regulation of gene expression during differentiation and cell fate determination, as demonstrated in myoblasts and embryonic stem cells [39]. Although the function of SEZ6L is not well understood, it has been shown both in mice and in vitro that this protein is almost exclusively processed by β-site APP cleaving enzyme (BACE) [40]; BACE1 and BACE2 are abundantly expressed in human placentae and are upregulated in pregnancies complicated with preeclampsia [41]. Lastly, *Rnf222* is also a protein-coding gene; however, no functional description has been found.

Although information on these genes in placentae is currently limited, it must be noted that a large number of placental genes and related phenotypes remain uncharacterized. Recent efforts to systematically identify the genes required for normal embryogenesis are still unravelling many previously underappreciated placental defects [42]. Thus, our findings might represent novel targets that could be implicated in the pathophysiology of maternal obesity and associated adverse outcomes in the offspring. Moreover, additional genes might have been identified if a larger sample size was used in the RNA-seq analysis.

When comparing both timepoints, most transcripts exhibited a different expression pattern, including *Muc15*, *Cnn1*, and *Acta2* which were not affected by obesity at E13. This could be due to low functional relevance of these transcripts at mid-pregnancy rather than absence of alterations in related cellular processes, as illustrated by low *Cnn1* mRNA levels in our analysis at E13 compared to E19 (data not shown). In fact, the mouse placenta undergoes a transcriptome transition from the “development phase” of organogenesis to the “mature phase” at mid-pregnancy [43].

Thus, we next used the IPA data to identify genes which were previously shown to be both highly expressed at mid-pregnancy and pivotal to spiral artery remodeling and formation of fetal blood vessels in the LZ. *Hand1*, which is required for trophoblast giant cell (TGC) differentiation [44], was downregulated in placentae of obese dams. However, maternal obesity had no effect on the expression of *Prl2c2*, a marker of TGC that line maternal blood canal spaces and spiral arteries in the definitive placenta [45]. Considering the complexity of spiral artery remodeling and the limited number of transcripts that were analyzed here, it remains to be established whether the alterations occur only later in development or if other mechanisms are involved.

On the other hand, the growth factor *Pdgfb* was downregulated in response to obesity at E13 and could be a relevant link to other molecular and phenotypic observations in our model. It has been shown that *Pdgfb*-deficient placentae exhibit defective labyrinthine development, with alterations in fetal blood vessel structure and reduced numbers of pericytes from mid-pregnancy until term, leading to growth restriction in PDGFB −/− embryos [29]. Here, lower expression of the pericyte marker *Acta2* was detected by RNA-seq and confirmed by qPCR in obese placentae at E19. In addition, defects in LZ morphology and FGR were observed in response to maternal obesity at E13 and persisted until E19.

As shown by our immunofluorescence staining, male and female placentae from Obese dams exhibited reduced LZ area, which is the primary site of gas, nutrient and waste exchange between the maternal and fetal circulations in the mouse [28], and a decrease in the proportion of fetal blood vessels within the LZ was also evident in females. This is further corroborated by recent evidence of lower vascularity in placentae of high fat diet-fed dams both at mid-pregnancy and near term, which was associated with placental transcriptome alterations in early stages of development and FGR [17]. In addition, it has been suggested that defects in placental villi vasculature seen in obese human pregnancies could be partly due to obesity-associated tissue hypoxia [46], which is also consistent with our model [20].

Next, we used IPA to investigate the mechanism behind this reduction in LZ area. Cellular growth and proliferation were pointed out as the main molecular and cellular functions affected by obesity. Surprisingly, however, maternal obesity had no effect on the number of Ki67-positive cells in the placenta. Abnormalities in placental size are often associated with disruption of cellular growth and/or apoptosis [47–49]. Thus, it is possible that other mechanisms such as cell death could explain our results, although limitations to our analysis, which was not zone-specific, cannot be discounted. In this regard, it has been shown in a mouse model of high fat diet-induced obesity through phosphohistone H3 staining that the proliferating cells in placenta are mostly restricted to the labyrinthine layer and appear reduced in response to obesity within this region [50].

Despite these morphological disturbances, changes in tissue structure that are expected to occur from mid-pregnancy until term seemed preserved in obese placentae. The LZ is well-reported to expand as pregnancy progresses, so that the transport capacity of the placenta can meet the nutrient demands of the growing fetus [50–52]. Accordingly, we found that labyrinth area increased between E13 and E19, irrespective of maternal diet.

We also observed significant differences in placentae which are specific to fetal sex. Female placentae were smaller than male counterparts at all timepoints and maternal conditions, which is consistent with observations from both human cohorts [53] and studies in mice [19, 54]. Moreover, we found sex differences in a subset of genes, with females exhibiting slightly increased expression. Similarly, global transcriptomic analysis in normal full-term human placentae revealed higher overall mRNA levels in females compared to males [55]. Sexual dimorphism in the context of developmental programming is increasingly commonly reported [56]. How these relate to sex-specific responses of the placenta to a suboptimal environment remain to be determined.

Overall, we have shown through genome-wide analysis that maternal obesity induces a dysregulation of transcripts and pathway interactions related to placental vasculature and structure. FGR, as well as changes in placental morphology and a gene expression signature associated with impaired labyrinthine development, were detectable at mid-pregnancy, suggesting an enduring negative effect of maternal obesity over these processes. The LZ is the exchange region of the murine placenta and reductions in its size and vasculature may impair the transport of nutrients from the maternal circulation to the developing fetus, thus restricting its growth. Disruption of placental structure could thus represent an important factor contributing to the development of FGR in pregnancies complicated by maternal obesity. Moreover, novel targets were revealed by our RNA-seq analysis. Characterizing their functional roles in the placenta will help us better understand the processes mediating the effects of maternal obesity on offspring outcomes and potentially inform suitable interventions.

## Supplementary information

Supplementary Figure S1

Supplementary Figure S2

Supplementary Figure S3

Supplementary Figure S4

Supplementary Table S1

Supplementary Table S2
