# Supplementary material for: Impact of maternal obesity on placental transcriptome and morphology associated with fetal growth restriction in mice
Source: Int J Obes (Lond). 2020 Mar 13;44(5):1087–96. doi: 10.1038/s41366-020-0561-3 (PMC7188669; doi:10.1038/s41366-020-0561-3)
Supplement: Supplementary file 1 — Supplementary Figure S1 [file 41366_2020_561_MOESM1_ESM.docx]

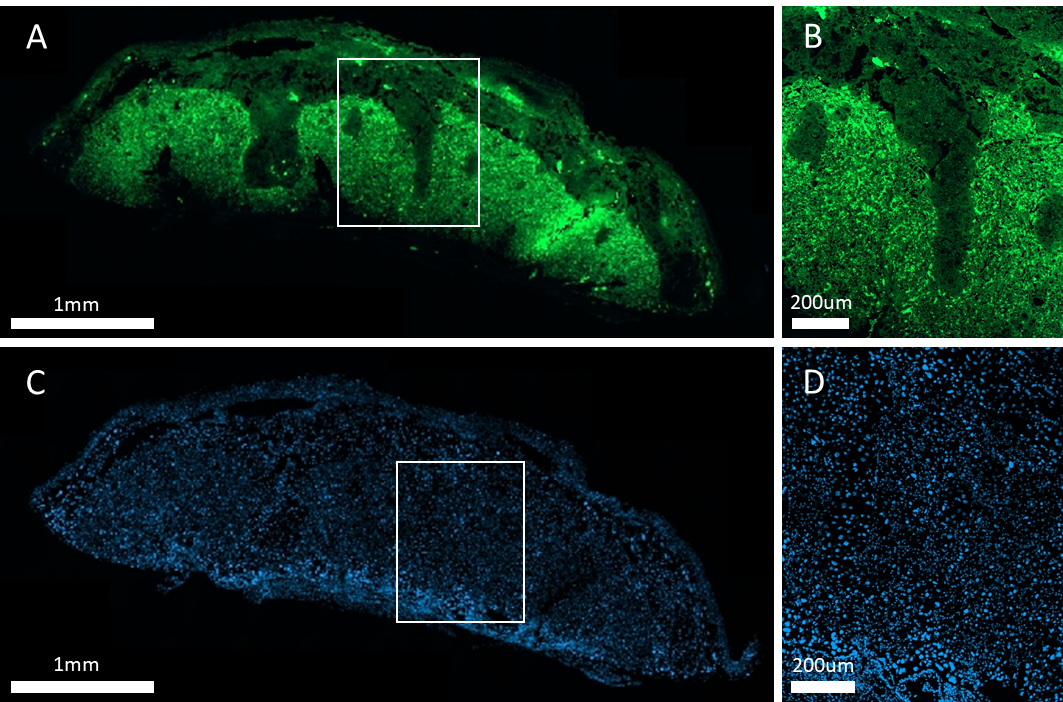


# **Supplementary Figure S1.** (A, B) Representative images illustrating need to quench autofluorescence when immunostaining formalin-fixed placentae. Placenta section subjected to antigen retrieval, blocked with 3% goat serum and then incubated in 1.5% goat serum in TBST only - no primary or secondary antibodies applied. Section was then mounted without DAPI staining or treatment with Vector TrueVIEW Autofluorescence Quenching Kit​. Therefore, all fluorescence seen is autofluorescence. (C, D) Negative control of immunofluorescent staining. Placenta section taken through full staining protocol as described, except with primary antibody incubation omitted. Staining shows DAPI (blue) but no evidence of non-specific staining by secondary antibody (green).
