# Supplementary material for: Impact of maternal obesity on placental transcriptome and morphology associated with fetal growth restriction in mice
Source: Int J Obes (Lond). 2020 Mar 13;44(5):1087–96. doi: 10.1038/s41366-020-0561-3 (PMC7188669; doi:10.1038/s41366-020-0561-3)
Supplement: Supplementary file 2 — Supplementary Figure S2 [file 41366_2020_561_MOESM2_ESM.docx]

**
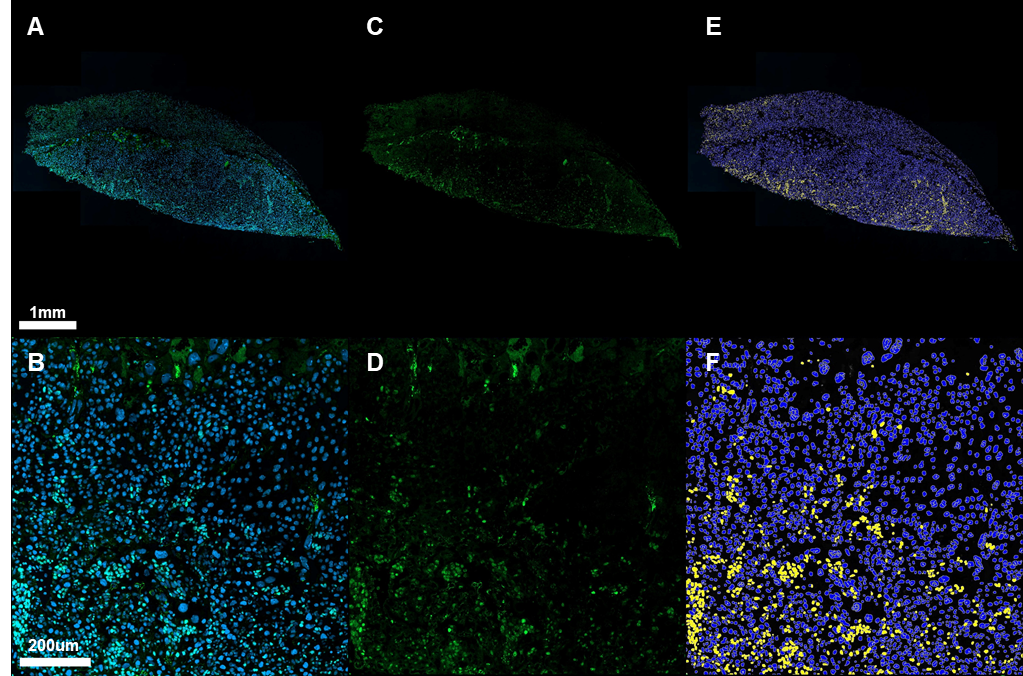
**

# **Supplementary Figure S2.** Immunofluorescent staining of Ki67 and analysis of cell proliferation. (A, B) Placental section stained for Ki67 (green) and DAPI (blue), or (C, D) Ki67 only; (E, F) HALO analysis output showing Ki67-negative (purple) and positive (yellow) nuclei.
