# Supplementary material for: Impact of maternal obesity on placental transcriptome and morphology associated with fetal growth restriction in mice
Source: Int J Obes (Lond). 2020 Mar 13;44(5):1087–96. doi: 10.1038/s41366-020-0561-3 (PMC7188669; doi:10.1038/s41366-020-0561-3)
Supplement: Supplementary file 3 — Supplementary Figure S3 [file 41366_2020_561_MOESM3_ESM.docx]

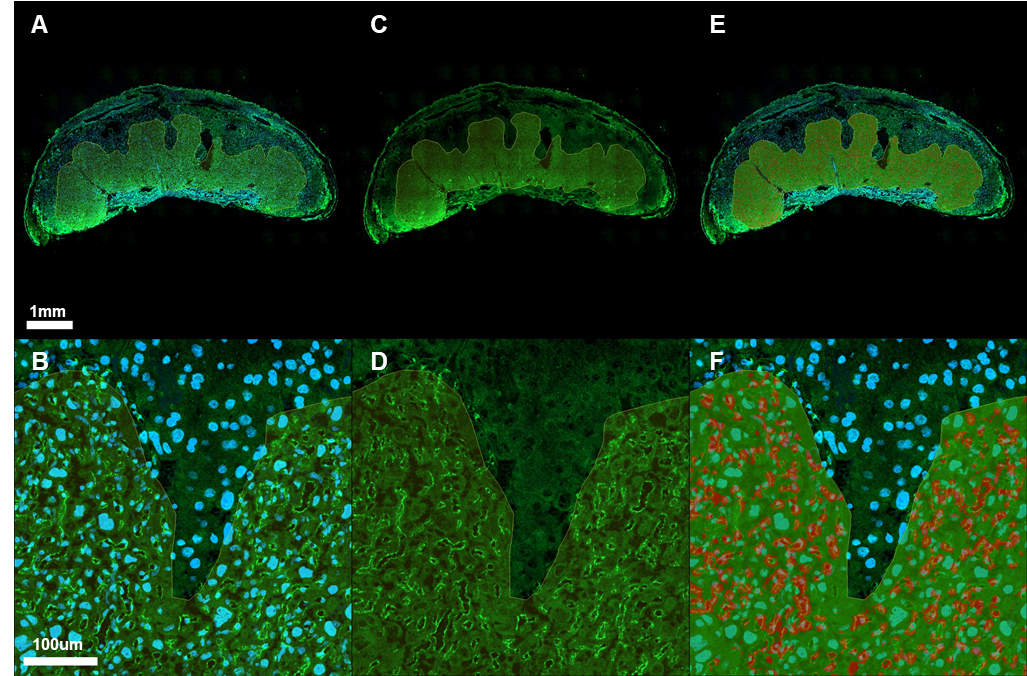


# **Supplementary Figure S3.** Immunofluorescent staining of CD31 and analysis of labyrinth fetal vasculature. **(A, B)** Placental section stained for CD31 (green) and DAPI (blue), or **(C, D)** CD31 only; **(E, F)** HALO analysis output showing regions of the labyrinth zone classified as fetal blood vessels (red) or other tissue (green).
