# Supplementary material for: Impact of maternal obesity on placental transcriptome and morphology associated with fetal growth restriction in mice
Source: Int J Obes (Lond). 2020 Mar 13;44(5):1087–96. doi: 10.1038/s41366-020-0561-3 (PMC7188669; doi:10.1038/s41366-020-0561-3)
Supplement: Supplementary file 4 — Supplementary Figure S4 [file 41366_2020_561_MOESM4_ESM.docx]

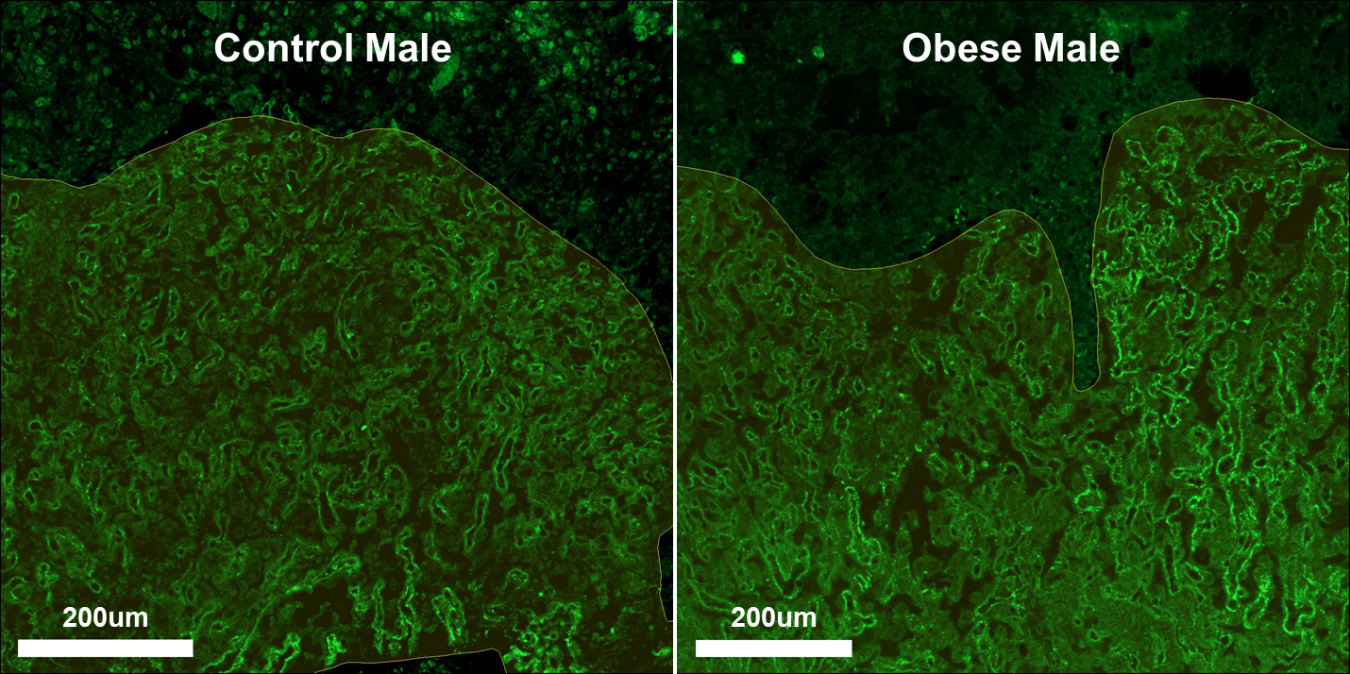


# **Supplementary Figure S4.** Immunofluorescent staining of CD31 in male placentae. The proportion of fetal capillaries within the labyrinth zone of male placentae was not affected by maternal obesity or gestational age, determined by two-way ANOVA analysis.
