# Supplementary material for: Impact of maternal obesity on placental transcriptome and morphology associated with fetal growth restriction in mice
Source: Int J Obes (Lond). 2020 Mar 13;44(5):1087–96. doi: 10.1038/s41366-020-0561-3 (PMC7188669; doi:10.1038/s41366-020-0561-3)
Supplement: Supplementary file 5 — Supplementary Table S1 [file 41366_2020_561_MOESM5_ESM.docx]

**Supplementary Table S1.** Specific sequences of primer pairs

| **Gene symbol** | **Forward primer sequence (5' to 3')** | **Reverse primer sequence (5' to 3')** |
| --- | --- | --- |
| *Acta2* | AATGTCCCCGCCATGTATGTG | CCATCTCCAGAGTCCAGCACA |
| *Cnn1* | TGCGCTTGTCTGTGTCATCT | TCTGGGCCAGCTTGTTCTTT |
| *Gabrd* | TGCCTGGTTCCATGATGTGAC | TACTTGGCGAGGTCCATGTCA |
| *Gapdh* | CAACGGGAAGCCCATCAC | GCCTCACCCCATTTGATGTT |
| *Hand1* | AGATCAAGACTCTGCGCCTG | TGCTGAGGCAACTCCCTTTTC |
| *Muc15* | CCCAAATACATCAGACACCCCA | TCTGTTTTCCGTTGTCCACACA |
| *Nup210* | TCCGTGTGTTGGACTTCTACA | ATGTGGCCGTGTAGTTGTCTA |
| *Pdgfb* | GGAGTCGGCATGAATCGCT | CAGCCCCATCTTCATCTACGG |
| *Pi15* | ATCATAATCAAGTCCGGGGCA | AGAAGGTCCATGGTCCCAAAT |
| *Pmm1* | ATCCGGGAGAAGTTTGTGGAA | GCTGTCTTCATCCAGGCTGTC |
| *Prl2c2* | GGCTCAGAGACAAAAGCCCC | GATCGTCCAGAGGGCTTTCC |
| *Rnf222* | TGAAGAATGCTCGGACATGGC | TCTCATAGCACACAGGGCACT |
| *Sdha* | TTCCGTGTGGGGAGTGTATTG | ATTCTGCAGCTCCAGGGTCTC |
| *Sez6l* | GGCACCACCATCCAGTACACC | CCAGGGACTCCTCAGAGACACA |
| *Sfrp4* | CCAATTCCTCCTGCCAGTGT | GAAGCATCATCCTTGAACGCC |
| *Ywhaz* | GAAAAGTTCTTGATCCCCAATGC | TGTGACTGGTCCACAATTCCTT |
