# Supplementary material for: Impact of maternal obesity on placental transcriptome and morphology associated with fetal growth restriction in mice
Source: Int J Obes (Lond). 2020 Mar 13;44(5):1087–96. doi: 10.1038/s41366-020-0561-3 (PMC7188669; doi:10.1038/s41366-020-0561-3)
Supplement: Supplementary file 6 — Supplementary Table S2 [file 41366_2020_561_MOESM6_ESM.docx]

**Supplementary Table S2.** RNA-seq identification of differentially expressed genes between Control (n=2) and Obese (n=3) male placentae at E19 (*P* < 0.05 threshold)

| **Ensembl ID** | **Gene name** | **logFC^a^** | **logCPM^b^** | ***P*-value** | **FDR^c^** |
| --- | --- | --- | --- | --- | --- |
| ENSMUSG00000067780 | *Pi15* | 1.789 | 2.8400 | 1.24E-08 | 0.0002 |
| ENSMUSG00000050808 | *Muc15* | -1.421 | 1.8371 | 2.40E-07 | 0.0015 |
| ENSMUSG00000046490 | *Rnf222* | -1.181 | 2.6460 | 8.62E-07 | 0.0035 |
| ENSMUSG00000030091 | *Nup210* | 0.718 | 5.0170 | 2.36E-06 | 0.0071 |
| ENSMUSG00000035783 | *Acta2* | -0.644 | 6.5599 | 3.88E-06 | 0.0094 |
| ENSMUSG00000058153 | *Sez6l* | 0.731 | 5.9094 | 5.46E-06 | 0.0110 |
| ENSMUSG00000001349 | *Cnn1* | -1.508 | 1.8182 | 1.19E-05 | 0.0206 |
| ENSMUSG00000029054 | *Gabrd* | 1.122 | 2.5846 | 1.80E-05 | 0.0272 |
| ENSMUSG00000021319 | *Sfrp4* | 0.765 | 5.7352 | 3.67E-05 | 0.0493 |
| ENSMUSG00000063011 | *Msln* | 0.644 | 6.4379 | 5.23E-05 | 0.0593 |
| ENSMUSG00000032085 | *Tagln* | -0.682 | 5.0760 | 5.40E-05 | 0.0593 |
| ENSMUSG00000020646 | *Mboat2* | 0.661 | 4.6138 | 0.0001 | 0.1342 |
| ENSMUSG00000022156 | *Gzme* | 0.979 | 2.6734 | 0.0002 | 0.1570 |
| ENSMUSG00000040284 | *Gzmg* | 0.997 | 4.0244 | 0.0002 | 0.1929 |
| ENSMUSG00000037216 | *Lipt1* | -0.915 | 2.2253 | 0.0003 | 0.2070 |
| ENSMUSG00000030111 | *A2m* | 0.692 | 8.3417 | 0.0003 | 0.2070 |
| ENSMUSG00000022309 | *Angpt1* | -0.863 | 2.5204 | 0.0003 | 0.2267 |
| ENSMUSG00000058152 | *Chsy3* | 1.000 | 2.5746 | 0.0003 | 0.2267 |
| ENSMUSG00000091898 | *Tnnc1* | -1.034 | 3.2519 | 0.0004 | 0.2686 |
| ENSMUSG00000000489 | *Pdgfb* | -0.564 | 6.4354 | 0.0006 | 0.3609 |
| ENSMUSG00000040612 | *Ildr2* | 0.711 | 4.0982 | 0.0006 | 0.3609 |
| ENSMUSG00000067818 | *Myl9* | -0.600 | 5.1425 | 0.0007 | 0.3814 |
| ENSMUSG00000018830 | *Myh11* | -0.535 | 5.7143 | 0.0009 | 0.4925 |
| ENSMUSG00000030162 | *Olr1* | 0.484 | 6.4898 | 0.0010 | 0.4925 |
| ENSMUSG00000070348 | *Ccnd1* | -0.574 | 4.5274 | 0.0013 | 0.5925 |
| ENSMUSG00000040808 | *S100g* | -0.528 | 7.4067 | 0.0013 | 0.5925 |
| ENSMUSG00000031403 | *Dkc1* | -0.772 | 2.3043 | 0.0014 | 0.6448 |
| ENSMUSG00000038463 | *Olfml2b* | -0.690 | 2.9904 | 0.0015 | 0.6448 |
| ENSMUSG00000018486 | *Wnt9b* | -0.851 | 2.0669 | 0.0016 | 0.6448 |
| ENSMUSG00000019256 | *Ahr* | -0.804 | 2.7284 | 0.0016 | 0.6448 |
| ENSMUSG00000091618 | *H60c* | -0.970 | 2.9737 | 0.0018 | 0.6723 |
| ENSMUSG00000007682 | *Dio2* | -0.819 | 3.5829 | 0.0018 | 0.6723 |
| ENSMUSG00000058794 | *Nfe2* | -0.882 | 1.9289 | 0.0022 | 0.7942 |
| ENSMUSG00000041986 | *Elmod1* | -0.632 | 2.9604 | 0.0023 | 0.8126 |
| ENSMUSG00000043366 | *Olfr78* | -0.990 | 1.4228 | 0.0024 | 0.8126 |
| ENSMUSG00000020340 | *Cyfip2* | 0.494 | 4.9603 | 0.0025 | 0.8284 |
| ENSMUSG00000049929 | *Lpar4* | -0.951 | 2.5623 | 0.0025 | 0.8284 |
| ENSMUSG00000059336 | *Slc14a1* | 0.765 | 3.5526 | 0.0027 | 0.8284 |
| ENSMUSG00000007655 | *Cav1* | -0.409 | 6.5378 | 0.0027 | 0.8284 |
| ENSMUSG00000022792 | *Yars2* | -0.593 | 3.3690 | 0.0030 | 0.9126 |
| ENSMUSG00000020925 | *Ccdc43* | -0.464 | 4.7011 | 0.0033 | 0.9843 |
| ENSMUSG00000001029 | *Icam2* | -0.588 | 3.2614 | 0.0036 | 0.9999 |
| ENSMUSG00000025650 | *Col7a1* | 0.854 | 3.5967 | 0.0037 | 0.9999 |
| ENSMUSG00000040936 | *Ulk4* | 0.887 | 1.9216 | 0.0037 | 0.9999 |
| ENSMUSG00000079186 | *Gzmc* | 0.838 | 3.2582 | 0.0041 | 0.9999 |
| ENSMUSG00000046805 | *Mpeg1* | 0.686 | 3.7515 | 0.0041 | 0.9999 |
| ENSMUSG00000021696 | *Elovl7* | -0.827 | 1.9279 | 0.0042 | 0.9999 |
| ENSMUSG00000059430 | *Actg2* | -0.722 | 3.8707 | 0.0042 | 0.9999 |
| ENSMUSG00000020143 | *Dock2* | 0.856 | 2.2588 | 0.0044 | 0.9999 |
| ENSMUSG00000056972 | *Magel2* | -0.744 | 2.3058 | 0.0044 | 0.9999 |
| ENSMUSG00000025467 | *Prap1* | 1.907 | 4.3370 | 0.0044 | 0.9999 |
| ENSMUSG00000020272 | *Stk10* | 0.443 | 4.9957 | 0.0046 | 0.9999 |
| ENSMUSG00000055675 | *Kbtbd11* | 0.842 | 2.2024 | 0.0046 | 0.9999 |
| ENSMUSG00000006221 | *Hspb7* | 0.522 | 4.5717 | 0.0053 | 0.9999 |
| ENSMUSG00000031089 | *Slc6a14* | -0.623 | 2.8546 | 0.0054 | 0.9999 |
| ENSMUSG00000028927 | *Padi2* | 0.508 | 6.8603 | 0.0055 | 0.9999 |
| ENSMUSG00000029096 | *Htra3* | 0.448 | 7.4407 | 0.0058 | 0.9999 |
| ENSMUSG00000078349 | *AW011738* | 0.962 | 2.6690 | 0.0062 | 0.9999 |
| ENSMUSG00000079436 | *Kcnj13* | -0.793 | 2.5724 | 0.0067 | 0.9999 |
| ENSMUSG00000036875 | *Dna2* | 0.530 | 5.0901 | 0.0067 | 0.9999 |
| ENSMUSG00000014592 | *Camta1* | 0.673 | 3.6334 | 0.0067 | 0.9999 |
| ENSMUSG00000063952 | *Brpf3* | 0.523 | 4.7994 | 0.0067 | 0.9999 |
| ENSMUSG00000045348 | *Nyap1* | 0.635 | 3.2251 | 0.0068 | 0.9999 |
| ENSMUSG00000058883 | *Zfp708* | -0.726 | 1.9463 | 0.0068 | 0.9999 |
| ENSMUSG00000063179 | *Pstk* | -0.695 | 2.3140 | 0.0069 | 0.9999 |
| ENSMUSG00000021903 | *Galnt15* | 0.639 | 3.3354 | 0.0069 | 0.9999 |
| ENSMUSG00000033446 | *Lpar6* | -0.489 | 3.9658 | 0.0071 | 0.9999 |
| ENSMUSG00000044229 | *Nxpe4* | 0.851 | 1.7848 | 0.0072 | 0.9999 |
| ENSMUSG00000004730 | *Adgre1* | 1.106 | 2.7943 | 0.0072 | 0.9999 |
| ENSMUSG00000036777 | *Anln* | -0.644 | 2.7756 | 0.0073 | 0.9999 |
| ENSMUSG00000020473 | *Aebp1* | 0.427 | 6.0542 | 0.0077 | 0.9999 |
| ENSMUSG00000030727 | *Rabep2* | 0.685 | 2.8100 | 0.0078 | 0.9999 |
| ENSMUSG00000039410 | *Prdm16* | 0.737 | 2.2401 | 0.0078 | 0.9999 |
| ENSMUSG00000026566 | *Mpzl1* | 0.364 | 6.4599 | 0.0082 | 0.9999 |
| ENSMUSG00000021390 | *Ogn* | -0.781 | 2.8129 | 0.0082 | 0.9999 |
| ENSMUSG00000042249 | *Adrbk2* | 0.747 | 2.3311 | 0.0083 | 0.9999 |
| ENSMUSG00000028019 | *Pdgfc* | -0.622 | 2.9211 | 0.0083 | 0.9999 |
| ENSMUSG00000032281 | *Acsbg1* | 0.548 | 3.3007 | 0.0085 | 0.9999 |
| ENSMUSG00000027869 | *Hsd3b6* | -0.896 | 1.8636 | 0.0086 | 0.9999 |
| ENSMUSG00000039470 | *Zdhhc2* | -0.507 | 3.7876 | 0.0086 | 0.9999 |
| ENSMUSG00000039458 | *Mtmr12* | -0.368 | 6.2885 | 0.0087 | 0.9999 |
| ENSMUSG00000048070 | *Pirt* | 0.664 | 2.3793 | 0.0088 | 0.9999 |
| ENSMUSG00000090394 | *4930523C07Rik* | -0.414 | 4.7526 | 0.0090 | 0.9999 |
| ENSMUSG00000004655 | *Aqp1* | 0.417 | 7.7524 | 0.0092 | 0.9999 |
| ENSMUSG00000054342 | *Kcnn4* | 0.691 | 2.6125 | 0.0092 | 0.9999 |
| ENSMUSG00000027800 | *Tm4sf1* | -0.545 | 4.4363 | 0.0092 | 0.9999 |
| ENSMUSG00000021451 | *Sema4d* | 0.456 | 4.4112 | 0.0092 | 0.9999 |
| ENSMUSG00000000305 | *Cdh4* | 0.711 | 2.7365 | 0.0095 | 0.9999 |
| ENSMUSG00000027313 | *Chac1* | -0.853 | 1.6873 | 0.0099 | 0.9999 |
| ENSMUSG00000058886 | *Deaf1* | 0.489 | 4.0557 | 0.0100 | 0.9999 |
| ENSMUSG00000033114 | *Slc35d2* | -0.625 | 2.8345 | 0.0104 | 0.9999 |
| ENSMUSG00000062184 | *Hs6st2* | -0.757 | 1.7432 | 0.0105 | 0.9999 |
| ENSMUSG00000097151 | *Gm26514* | 0.688 | 2.4441 | 0.0109 | 0.9999 |
| ENSMUSG00000005846 | *Rsl1d1* | -0.346 | 6.0245 | 0.0112 | 0.9999 |
| ENSMUSG00000045954 | *Sdpr* | -0.523 | 5.1272 | 0.0115 | 0.9999 |
| ENSMUSG00000040249 | *Lrp1* | 0.408 | 8.9708 | 0.0116 | 0.9999 |
| ENSMUSG00000056055 | *Sag* | 0.743 | 1.9025 | 0.0116 | 0.9999 |
| ENSMUSG00000063430 | *Wscd2* | 0.650 | 3.3218 | 0.0116 | 0.9999 |
| ENSMUSG00000075273 | *Ttc30b* | -0.539 | 2.9878 | 0.0120 | 0.9999 |
| ENSMUSG00000015441 | *Gzmf* | 0.665 | 4.5101 | 0.0124 | 0.9999 |
| ENSMUSG00000022120 | *Rnf219* | -0.560 | 2.8041 | 0.0125 | 0.9999 |
| ENSMUSG00000064368 | *mt-Nd6* | -0.400 | 4.8917 | 0.0127 | 0.9999 |
| ENSMUSG00000028631 | *Kcnq4* | 0.645 | 2.7583 | 0.0128 | 0.9999 |
| ENSMUSG00000020888 | *Dvl2* | 0.501 | 3.5495 | 0.0131 | 0.9999 |
| ENSMUSG00000016356 | *Col20a1* | 0.705 | 2.9365 | 0.0132 | 0.9999 |
| ENSMUSG00000024855 | *Pacs1* | 0.446 | 4.2393 | 0.0133 | 0.9999 |
| ENSMUSG00000079092 | *Prl2c2* | 0.532 | 4.8438 | 0.0135 | 0.9999 |
| ENSMUSG00000016024 | *Lbp* | 0.469 | 5.6519 | 0.0138 | 0.9999 |
| ENSMUSG00000072640 | *Lyrm9* | -0.572 | 3.2049 | 0.0138 | 0.9999 |
| ENSMUSG00000031790 | *Mmp15* | 0.336 | 6.9553 | 0.0138 | 0.9999 |
| ENSMUSG00000064080 | *Fbln2* | 0.310 | 6.4056 | 0.0139 | 0.9999 |
| ENSMUSG00000043419 | *Chd3os* | -0.562 | 2.7407 | 0.0140 | 0.9999 |
| ENSMUSG00000030064 | *Frmd4b* | -0.394 | 5.7053 | 0.0142 | 0.9999 |
| ENSMUSG00000032135 | *Mcam* | -0.347 | 6.2316 | 0.0147 | 0.9999 |
| ENSMUSG00000030256 | *Bhlhe41* | 0.481 | 5.7732 | 0.0148 | 0.9999 |
| ENSMUSG00000034194 | *R3hcc1* | -0.506 | 3.0612 | 0.0150 | 0.9999 |
| ENSMUSG00000064267 | *Hvcn1* | 0.425 | 4.3934 | 0.0152 | 0.9999 |
| ENSMUSG00000022123 | *Scel* | -0.410 | 6.3927 | 0.0152 | 0.9999 |
| ENSMUSG00000038843 | *Gcnt1* | -0.448 | 7.2621 | 0.0154 | 0.9999 |
| ENSMUSG00000013483 | *Card14* | 0.796 | 2.2861 | 0.0154 | 0.9999 |
| ENSMUSG00000026036 | *Nif3l1* | -0.478 | 4.0332 | 0.0154 | 0.9999 |
| ENSMUSG00000054850 | *Smim10l2a* | 0.598 | 2.7411 | 0.0155 | 0.9999 |
| ENSMUSG00000006711 | *D130043K22Rik* | 0.833 | 1.8120 | 0.0156 | 0.9999 |
| ENSMUSG00000035407 | *Kank4* | -0.473 | 3.3168 | 0.0158 | 0.9999 |
| ENSMUSG00000053062 | *Jam2* | 0.544 | 4.6774 | 0.0158 | 0.9999 |
| ENSMUSG00000073209 | *Klf14* | -0.587 | 2.9669 | 0.0159 | 0.9999 |
| ENSMUSG00000033985 | *Tesk2* | -0.480 | 5.0700 | 0.0161 | 0.9999 |
| ENSMUSG00000046550 | *Spin2c* | -0.778 | 1.4481 | 0.0161 | 0.9999 |
| ENSMUSG00000049093 | *Il23r* | 0.829 | 2.4453 | 0.0162 | 0.9999 |
| ENSMUSG00000017002 | *Slpi* | 0.570 | 4.0847 | 0.0162 | 0.9999 |
| ENSMUSG00000028328 | *Tmod1* | 0.593 | 3.6993 | 0.0163 | 0.9999 |
| ENSMUSG00000097333 | *Zfp87* | 0.498 | 3.3985 | 0.0167 | 0.9999 |
| ENSMUSG00000027233 | *Patl2* | -0.511 | 2.6919 | 0.0172 | 0.9999 |
| ENSMUSG00000074211 | *Sdhaf1* | 0.690 | 2.4539 | 0.0174 | 0.9999 |
| ENSMUSG00000031897 | *Psmb10* | -0.538 | 2.9243 | 0.0174 | 0.9999 |
| ENSMUSG00000003617 | *Cp* | -0.471 | 5.1827 | 0.0176 | 0.9999 |
| ENSMUSG00000061132 | *Blnk* | -0.689 | 1.6633 | 0.0182 | 0.9999 |
| ENSMUSG00000027408 | *Cpxm1* | 0.403 | 5.7359 | 0.0183 | 0.9999 |
| ENSMUSG00000047123 | *Ticam1* | 0.488 | 4.0960 | 0.0186 | 0.9999 |
| ENSMUSG00000038203 | *Hoxa13* | -0.554 | 2.7186 | 0.0189 | 0.9999 |
| ENSMUSG00000041577 | *Prelp* | 0.385 | 4.8800 | 0.0190 | 0.9999 |
| ENSMUSG00000087141 | *Plcxd2* | -0.471 | 5.6710 | 0.0191 | 0.9999 |
| ENSMUSG00000026259 | *Ngef* | 0.775 | 1.7424 | 0.0192 | 0.9999 |
| ENSMUSG00000064360 | *mt-Nd3* | -0.316 | 7.5166 | 0.0196 | 0.9999 |
| ENSMUSG00000034450 | *Gulo* | -0.484 | 4.5116 | 0.0199 | 0.9999 |
| ENSMUSG00000039007 | *Cpq* | 0.515 | 2.8951 | 0.0199 | 0.9999 |
| ENSMUSG00000059256 | *Gzmd* | 0.645 | 4.1611 | 0.0201 | 0.9999 |
| ENSMUSG00000031538 | *Plat* | -0.393 | 5.5815 | 0.0202 | 0.9999 |
| ENSMUSG00000046152 | *Fut10* | -0.572 | 2.4185 | 0.0206 | 0.9999 |
| ENSMUSG00000061086 | *Myl4* | -0.557 | 5.0280 | 0.0207 | 0.9999 |
| ENSMUSG00000019848 | *Popdc3* | -0.600 | 3.8127 | 0.0207 | 0.9999 |
| ENSMUSG00000010154 | *Spire2* | -0.548 | 2.7100 | 0.0208 | 0.9999 |
| ENSMUSG00000051166 | *Eml5* | -0.479 | 3.7096 | 0.0211 | 0.9999 |
| ENSMUSG00000031965 | *Tbx20* | -0.599 | 4.0803 | 0.0211 | 0.9999 |
| ENSMUSG00000026271 | *Gpr35* | -0.554 | 3.2228 | 0.0212 | 0.9999 |
| ENSMUSG00000054435 | *Gimap4* | -0.515 | 2.5582 | 0.0214 | 0.9999 |
| ENSMUSG00000046865 | *Fbl* | -0.487 | 2.8605 | 0.0216 | 0.9999 |
| ENSMUSG00000062380 | *Tubb3* | -0.380 | 6.3187 | 0.0217 | 0.9999 |
| ENSMUSG00000025020 | *Slit1* | 0.343 | 6.4200 | 0.0218 | 0.9999 |
| ENSMUSG00000025235 | *Bbs4* | 0.473 | 3.5949 | 0.0219 | 0.9999 |
| ENSMUSG00000023961 | *Enpp4* | 0.528 | 2.7422 | 0.0221 | 0.9999 |
| ENSMUSG00000029675 | *Eln* | -0.555 | 4.2181 | 0.0223 | 0.9999 |
| ENSMUSG00000032028 | *Nxpe2* | -0.742 | 1.3877 | 0.0223 | 0.9999 |
| ENSMUSG00000032311 | *Nrg4* | 0.639 | 2.3599 | 0.0223 | 0.9999 |
| ENSMUSG00000041889 | *Shisa4* | -0.496 | 3.0430 | 0.0225 | 0.9999 |
| ENSMUSG00000028763 | *Hspg2* | 0.346 | 8.6193 | 0.0225 | 0.9999 |
| ENSMUSG00000032356 | *Rasgrf1* | 0.707 | 2.0393 | 0.0227 | 0.9999 |
| ENSMUSG00000024589 | *Nedd4l* | 0.317 | 6.1969 | 0.0230 | 0.9999 |
| ENSMUSG00000061887 | *Ssbp3* | 0.408 | 4.7029 | 0.0232 | 0.9999 |
| ENSMUSG00000031937 | *Vstm5* | 0.710 | 2.5948 | 0.0233 | 0.9999 |
| ENSMUSG00000051839 | *Gypa* | 0.746 | 1.6828 | 0.0234 | 0.9999 |
| ENSMUSG00000015619 | *Gata3* | 0.367 | 4.7455 | 0.0236 | 0.9999 |
| ENSMUSG00000027297 | *Ltk* | -0.627 | 2.7487 | 0.0238 | 0.9999 |
| ENSMUSG00000064068 | *Mtx1* | -0.451 | 4.2704 | 0.0239 | 0.9999 |
| ENSMUSG00000055485 | *Soga1* | 0.349 | 5.6217 | 0.0242 | 0.9999 |
| ENSMUSG00000046574 | *Prr12* | 0.652 | 2.2123 | 0.0242 | 0.9999 |
| ENSMUSG00000046591 | *Ticrr* | 0.553 | 2.7393 | 0.0242 | 0.9999 |
| ENSMUSG00000049422 | *Chchd10* | 0.375 | 4.6970 | 0.0242 | 0.9999 |
| ENSMUSG00000004098 | *Col5a3* | 0.462 | 3.5545 | 0.0243 | 0.9999 |
| ENSMUSG00000025403 | *Shmt2* | 0.372 | 4.4405 | 0.0250 | 0.9999 |
| ENSMUSG00000055760 | *Gemin6* | 0.838 | 1.9768 | 0.0252 | 0.9999 |
| ENSMUSG00000028838 | *Extl1* | 0.385 | 4.7407 | 0.0254 | 0.9999 |
| ENSMUSG00000037321 | *Tap1* | 0.341 | 4.9792 | 0.0254 | 0.9999 |
| ENSMUSG00000024041 | *Cryaa* | 0.458 | 4.5264 | 0.0255 | 0.9999 |
| ENSMUSG00000037989 | *Wnk2* | 0.546 | 2.8682 | 0.0255 | 0.9999 |
| ENSMUSG00000026395 | *Ptprc* | 0.627 | 3.1270 | 0.0256 | 0.9999 |
| ENSMUSG00000033400 | *Agl* | 0.300 | 6.3430 | 0.0256 | 0.9999 |
| ENSMUSG00000021255 | *Esrrb* | -0.607 | 2.0993 | 0.0258 | 0.9999 |
| ENSMUSG00000086742 | *Gm16201* | 0.602 | 2.3217 | 0.0259 | 0.9999 |
| ENSMUSG00000033669 | *Zfp7* | -0.491 | 3.7405 | 0.0264 | 0.9999 |
| ENSMUSG00000026435 | *Slc45a3* | -0.436 | 5.2363 | 0.0265 | 0.9999 |
| ENSMUSG00000091405 | *Hist2h4* | 0.537 | 2.5927 | 0.0265 | 0.9999 |
| ENSMUSG00000075031 | *Hist1h2bb* | -0.665 | 1.7628 | 0.0267 | 0.9999 |
| ENSMUSG00000034110 | *Kctd7* | 0.645 | 2.2885 | 0.0268 | 0.9999 |
| ENSMUSG00000026582 | *Sele* | 0.409 | 5.3289 | 0.0269 | 0.9999 |
| ENSMUSG00000087166 | *L1td1* | -0.451 | 3.6540 | 0.0269 | 0.9999 |
| ENSMUSG00000030745 | *Il21r* | 0.785 | 3.0327 | 0.0271 | 0.9999 |
| ENSMUSG00000020701 | *Tmem132e* | 0.463 | 3.6122 | 0.0271 | 0.9999 |
| ENSMUSG00000020228 | *Helb* | 0.492 | 2.9104 | 0.0271 | 0.9999 |
| ENSMUSG00000048534 | *Amica1* | -0.436 | 4.1427 | 0.0275 | 0.9999 |
| ENSMUSG00000051228 | *Nyx* | -0.438 | 3.1828 | 0.0277 | 0.9999 |
| ENSMUSG00000021003 | *Galc* | 0.457 | 3.9799 | 0.0277 | 0.9999 |
| ENSMUSG00000062515 | *Fabp4* | 0.356 | 6.3198 | 0.0278 | 0.9999 |
| ENSMUSG00000097391 | *Mirg* | -0.371 | 4.9586 | 0.0279 | 0.9999 |
| ENSMUSG00000024935 | *Slc1a1* | -0.457 | 5.6688 | 0.0280 | 0.9999 |
| ENSMUSG00000045930 | *Clec14a* | -0.433 | 6.0325 | 0.0280 | 0.9999 |
| ENSMUSG00000032802 | *Srxn1* | -0.486 | 5.4678 | 0.0283 | 0.9999 |
| ENSMUSG00000025902 | *Sox17* | -0.464 | 3.7982 | 0.0283 | 0.9999 |
| ENSMUSG00000037490 | *Slc2a12* | -0.571 | 3.9018 | 0.0286 | 0.9999 |
| ENSMUSG00000025265 | *Fgd1* | 0.472 | 4.8533 | 0.0289 | 0.9999 |
| ENSMUSG00000067276 | *Capn6* | -0.468 | 3.5716 | 0.0295 | 0.9999 |
| ENSMUSG00000066278 | *Vps37b* | 0.420 | 3.8881 | 0.0298 | 0.9999 |
| ENSMUSG00000039233 | *Tbce* | -0.364 | 4.5520 | 0.0299 | 0.9999 |
| ENSMUSG00000024206 | *Rfx2* | -0.628 | 1.8290 | 0.0300 | 0.9999 |
| ENSMUSG00000021265 | *Slc25a29* | 0.553 | 3.4747 | 0.0301 | 0.9999 |
| ENSMUSG00000069743 | *Zfp820* | -0.769 | 1.9561 | 0.0302 | 0.9999 |
| ENSMUSG00000025037 | *Maoa* | -0.280 | 6.5543 | 0.0302 | 0.9999 |
| ENSMUSG00000022297 | *Fzd6* | -0.346 | 5.1393 | 0.0305 | 0.9999 |
| ENSMUSG00000017144 | *Rnd3* | -0.323 | 5.3957 | 0.0306 | 0.9999 |
| ENSMUSG00000022579 | *Gpihbp1* | -0.473 | 3.4574 | 0.0307 | 0.9999 |
| ENSMUSG00000002489 | *Tiam1* | 0.350 | 4.9536 | 0.0307 | 0.9999 |
| ENSMUSG00000050379 | *Sept6* | 0.455 | 4.4262 | 0.0311 | 0.9999 |
| ENSMUSG00000044700 | *Tmem201* | 0.427 | 3.5019 | 0.0317 | 0.9999 |
| ENSMUSG00000027978 | *Prss12* | -0.538 | 3.2231 | 0.0318 | 0.9999 |
| ENSMUSG00000039620 | *6430573F11Rik* | 0.655 | 2.1191 | 0.0320 | 0.9999 |
| ENSMUSG00000050608 | *Minos1* | 0.338 | 4.6287 | 0.0322 | 0.9999 |
| ENSMUSG00000026222 | *Sp100* | -0.558 | 2.6513 | 0.0326 | 0.9999 |
| ENSMUSG00000069601 | *Ank3* | -0.378 | 5.0405 | 0.0326 | 0.9999 |
| ENSMUSG00000034203 | *Chchd4* | -0.415 | 4.0244 | 0.0328 | 0.9999 |
| ENSMUSG00000046410 | *Kcnk6* | -0.338 | 4.8874 | 0.0329 | 0.9999 |
| ENSMUSG00000002028 | *Kmt2a* | 0.303 | 5.8575 | 0.0330 | 0.9999 |
| ENSMUSG00000037211 | *Spry1* | -0.579 | 3.5481 | 0.0335 | 0.9999 |
| ENSMUSG00000039167 | *Adgrl4* | -0.366 | 5.7987 | 0.0338 | 0.9999 |
| ENSMUSG00000087233 | *Gm43213* | 0.667 | 1.7704 | 0.0339 | 0.9999 |
| ENSMUSG00000038648 | *Creb3l2* | 0.293 | 6.8265 | 0.0339 | 0.9999 |
| ENSMUSG00000028581 | *Laptm5* | 0.617 | 2.5976 | 0.0339 | 0.9999 |
| ENSMUSG00000072762 | *4930522L14Rik* | -0.508 | 3.6745 | 0.0341 | 0.9999 |
| ENSMUSG00000038587 | *Akap12* | -0.340 | 6.1428 | 0.0342 | 0.9999 |
| ENSMUSG00000064356 | *mt-Atp8* | -0.401 | 4.8368 | 0.0348 | 0.9999 |
| ENSMUSG00000023951 | *Vegfa* | 0.314 | 6.8026 | 0.0350 | 0.9999 |
| ENSMUSG00000085334 | *Gm12940* | -0.414 | 3.3716 | 0.0351 | 0.9999 |
| ENSMUSG00000050022 | *Amz1* | 0.507 | 3.7947 | 0.0352 | 0.9999 |
| ENSMUSG00000022052 | *Ppp2r2a* | 0.407 | 4.3336 | 0.0352 | 0.9999 |
| ENSMUSG00000009585 | *Apobec3* | 0.621 | 1.9686 | 0.0354 | 0.9999 |
| ENSMUSG00000045948 | *Mrps12* | 0.455 | 3.2828 | 0.0354 | 0.9999 |
| ENSMUSG00000026532 | *Spta1* | 0.812 | 1.9859 | 0.0355 | 0.9999 |
| ENSMUSG00000005950 | *P2rx5* | 0.730 | 1.7749 | 0.0357 | 0.9999 |
| ENSMUSG00000003352 | *Cacnb3* | 0.467 | 4.2450 | 0.0357 | 0.9999 |
| ENSMUSG00000031131 | *Vgll1* | -0.451 | 3.0673 | 0.0359 | 0.9999 |
| ENSMUSG00000069763 | *Tmem100* | -0.356 | 5.4730 | 0.0359 | 0.9999 |
| ENSMUSG00000027611 | *Procr* | 0.405 | 8.0929 | 0.0359 | 0.9999 |
| ENSMUSG00000037625 | *Cldn11* | 0.421 | 5.0015 | 0.0360 | 0.9999 |
| ENSMUSG00000031749 | *St3gal2* | 0.395 | 4.2430 | 0.0361 | 0.9999 |
| ENSMUSG00000022818 | *Cyp2ab1* | 0.817 | 1.8131 | 0.0361 | 0.9999 |
| ENSMUSG00000025911 | *Adhfe1* | 0.542 | 2.4824 | 0.0363 | 0.9999 |
| ENSMUSG00000072945 | *Ripply1* | -0.506 | 3.8981 | 0.0366 | 0.9999 |
| ENSMUSG00000018263 | *Tbx5* | -0.610 | 1.7971 | 0.0366 | 0.9999 |
| ENSMUSG00000000340 | *Dbt* | -0.385 | 4.2178 | 0.0367 | 0.9999 |
| ENSMUSG00000022887 | *Masp1* | 0.392 | 6.1221 | 0.0367 | 0.9999 |
| ENSMUSG00000020453 | *Patz1* | -0.414 | 3.5752 | 0.0371 | 0.9999 |
| ENSMUSG00000051586 | *Mical3* | 0.285 | 6.6195 | 0.0371 | 0.9999 |
| ENSMUSG00000028864 | *Hgf* | -0.756 | 1.3570 | 0.0374 | 0.9999 |
| ENSMUSG00000074194 | *Zfp791* | 0.666 | 2.2521 | 0.0377 | 0.9999 |
| ENSMUSG00000026972 | *Arrdc1* | 0.482 | 2.9879 | 0.0377 | 0.9999 |
| ENSMUSG00000019467 | *Arhgef25* | 0.386 | 5.0094 | 0.0380 | 0.9999 |
| ENSMUSG00000000730 | *Dnmt3l* | -0.500 | 3.9605 | 0.0380 | 0.9999 |
| ENSMUSG00000022283 | *Pabpc1* | 0.287 | 6.9794 | 0.0385 | 0.9999 |
| ENSMUSG00000022044 | *Stmn4* | -0.675 | 1.4690 | 0.0385 | 0.9999 |
| ENSMUSG00000020654 | *Adcy3* | -0.471 | 2.8759 | 0.0386 | 0.9999 |
| ENSMUSG00000039345 | *Mettl22* | 0.570 | 2.0978 | 0.0387 | 0.9999 |
| ENSMUSG00000027667 | *Zfp639* | -0.311 | 5.1536 | 0.0389 | 0.9999 |
| ENSMUSG00000039542 | *Ncam1* | 0.338 | 5.4076 | 0.0390 | 0.9999 |
| ENSMUSG00000030279 | *C2cd5* | 0.395 | 4.0421 | 0.0393 | 0.9999 |
| ENSMUSG00000036040 | *Adamtsl2* | -0.752 | 1.3407 | 0.0394 | 0.9999 |
| ENSMUSG00000028883 | *Sema3a* | -0.509 | 3.3047 | 0.0396 | 0.9999 |
| ENSMUSG00000038147 | *Cd84* | 0.610 | 1.8656 | 0.0397 | 0.9999 |
| ENSMUSG00000036975 | *Tmem177* | -0.439 | 4.4116 | 0.0397 | 0.9999 |
| ENSMUSG00000034892 | *Rps29* | 0.293 | 6.1558 | 0.0398 | 0.9999 |
| ENSMUSG00000025856 | *Pdgfa* | -0.307 | 5.6842 | 0.0401 | 0.9999 |
| ENSMUSG00000003344 | *Btbd2* | 0.427 | 3.8042 | 0.0401 | 0.9999 |
| ENSMUSG00000028393 | *Alad* | 0.340 | 5.2874 | 0.0405 | 0.9999 |
| ENSMUSG00000035504 | *Reep6* | 0.478 | 3.4322 | 0.0407 | 0.9999 |
| ENSMUSG00000027514 | *Zbp1* | 0.611 | 1.9191 | 0.0407 | 0.9999 |
| ENSMUSG00000031697 | *Orc6* | -0.380 | 4.5083 | 0.0409 | 0.9999 |
| ENSMUSG00000054013 | *Tmem179* | 0.656 | 1.6969 | 0.0409 | 0.9999 |
| ENSMUSG00000025964 | *Adam23* | -0.371 | 5.0668 | 0.0411 | 0.9999 |
| ENSMUSG00000033419 | *Snap91* | -0.309 | 6.0103 | 0.0412 | 0.9999 |
| ENSMUSG00000047250 | *Ptgs1* | -0.321 | 5.4478 | 0.0415 | 0.9999 |
| ENSMUSG00000021136 | *Smoc1* | 0.375 | 5.6044 | 0.0416 | 0.9999 |
| ENSMUSG00000023885 | *Thbs2* | 0.285 | 7.1616 | 0.0417 | 0.9999 |
| ENSMUSG00000031765 | *Mt1* | 0.319 | 6.6248 | 0.0421 | 0.9999 |
| ENSMUSG00000027304 | *Rtf1* | -0.270 | 6.6805 | 0.0422 | 0.9999 |
| ENSMUSG00000011382 | *Dhdh* | -0.571 | 1.7606 | 0.0426 | 0.9999 |
| ENSMUSG00000031853 | *BC021891* | 0.455 | 4.2627 | 0.0426 | 0.9999 |
| ENSMUSG00000070699 | *Sars2* | 0.560 | 2.4558 | 0.0430 | 0.9999 |
| ENSMUSG00000042182 | *Bend6* | 0.440 | 3.1651 | 0.0435 | 0.9999 |
| ENSMUSG00000078532 | *Nkain1* | 0.555 | 2.0720 | 0.0436 | 0.9999 |
| ENSMUSG00000003865 | *Gys1* | 0.288 | 5.9195 | 0.0437 | 0.9999 |
| ENSMUSG00000030188 | *Magohb* | -0.577 | 1.8536 | 0.0440 | 0.9999 |
| ENSMUSG00000029722 | *Agfg2* | 0.409 | 3.4683 | 0.0441 | 0.9999 |
| ENSMUSG00000042590 | *Ipo11* | -0.301 | 5.0648 | 0.0442 | 0.9999 |
| ENSMUSG00000030513 | *Pcsk6* | 0.411 | 3.8044 | 0.0447 | 0.9999 |
| ENSMUSG00000050069 | *Grem2* | 0.481 | 3.4557 | 0.0447 | 0.9999 |
| ENSMUSG00000021461 | *Fancc* | -0.368 | 3.8796 | 0.0448 | 0.9999 |
| ENSMUSG00000074277 | *Phldb3* | 0.312 | 4.9649 | 0.0452 | 0.9999 |
| ENSMUSG00000029407 | *Uso1* | 0.307 | 6.2210 | 0.0452 | 0.9999 |
| ENSMUSG00000027674 | *Pex5l* | 0.509 | 2.5003 | 0.0452 | 0.9999 |
| ENSMUSG00000043943 | *Naalad2* | 0.558 | 2.3571 | 0.0453 | 0.9999 |
| ENSMUSG00000035032 | *Nek11* | 0.684 | 1.6691 | 0.0454 | 0.9999 |
| ENSMUSG00000020806 | *Rhbdf2* | 0.411 | 3.5014 | 0.0455 | 0.9999 |
| ENSMUSG00000038264 | *Sema7a* | 0.278 | 6.0758 | 0.0456 | 0.9999 |
| ENSMUSG00000046794 | *Ppp1r3b* | 0.307 | 5.6685 | 0.0457 | 0.9999 |
| ENSMUSG00000068196 | *Col8a1* | -0.641 | 2.2104 | 0.0458 | 0.9999 |
| ENSMUSG00000037563 | *Rps16* | 0.312 | 5.8213 | 0.0458 | 0.9999 |
| ENSMUSG00000042532 | *Golga7b* | 0.583 | 3.4505 | 0.0459 | 0.9999 |
| ENSMUSG00000021263 | *Degs2* | 0.506 | 4.5453 | 0.0460 | 0.9999 |
| ENSMUSG00000032350 | *Gclc* | -0.382 | 6.5594 | 0.0462 | 0.9999 |
| ENSMUSG00000023046 | *Igfbp6* | -0.523 | 4.9966 | 0.0463 | 0.9999 |
| ENSMUSG00000051098 | *Mblac2* | 0.748 | 1.6184 | 0.0465 | 0.9999 |
| ENSMUSG00000097451 | *Rian* | -0.311 | 8.3809 | 0.0471 | 0.9999 |
| ENSMUSG00000030123 | *Plxnd1* | 0.288 | 5.8411 | 0.0472 | 0.9999 |
| ENSMUSG00000048970 | *C1galt1c1* | -0.387 | 3.9035 | 0.0472 | 0.9999 |
| ENSMUSG00000042677 | *Zc3h12a* | 0.679 | 1.6270 | 0.0473 | 0.9999 |
| ENSMUSG00000017400 | *Stac2* | 0.510 | 2.2934 | 0.0473 | 0.9999 |
| ENSMUSG00000019986 | *Ahi1* | 0.420 | 3.3625 | 0.0473 | 0.9999 |
| ENSMUSG00000066894 | *Vsig10* | -0.311 | 5.0459 | 0.0474 | 0.9999 |
| ENSMUSG00000030223 | *Ptpro* | 0.540 | 2.7823 | 0.0476 | 0.9999 |
| ENSMUSG00000034687 | *Fras1* | 0.754 | 1.7890 | 0.0478 | 0.9999 |
| ENSMUSG00000062300 | *Nectin2* | 0.267 | 7.1835 | 0.0480 | 0.9999 |
| ENSMUSG00000039835 | *Nhsl1* | 0.331 | 4.5895 | 0.0480 | 0.9999 |
| ENSMUSG00000023009 | *Nckap5l* | 0.425 | 3.0062 | 0.0481 | 0.9999 |
| ENSMUSG00000016194 | *Hsd11b1* | -0.396 | 7.6893 | 0.0482 | 0.9999 |
| ENSMUSG00000035105 | *Egln3* | 0.461 | 5.7726 | 0.0484 | 0.9999 |
| ENSMUSG00000015405 | *Ace2* | -0.354 | 6.2956 | 0.0485 | 0.9999 |
| ENSMUSG00000062619 | *2310039H08Rik* | -0.670 | 1.4101 | 0.0486 | 0.9999 |
| ENSMUSG00000022747 | *St3gal6* | -0.349 | 6.2377 | 0.0486 | 0.9999 |
| ENSMUSG00000034639 | *Setmar* | -0.619 | 1.5578 | 0.0486 | 0.9999 |
| ENSMUSG00000040929 | *Rfx3* | -0.423 | 3.1907 | 0.0488 | 0.9999 |
| ENSMUSG00000020627 | *Klhl29* | 0.402 | 3.7629 | 0.0488 | 0.9999 |
| ENSMUSG00000039246 | *Lyplal1* | 0.595 | 1.8421 | 0.0489 | 0.9999 |
| ENSMUSG00000021432 | *Slc35b3* | 0.299 | 4.9485 | 0.0490 | 0.9999 |
| ENSMUSG00000024053 | *Emilin2* | 0.339 | 6.8385 | 0.0492 | 0.9999 |
| ENSMUSG00000041040 | *Fam117b* | 0.283 | 5.7854 | 0.0494 | 0.9999 |
| ENSMUSG00000048473 | *Sult6b2* | -0.667 | 1.3092 | 0.0495 | 0.9999 |
| ENSMUSG00000027397 | *Slc20a1* | -0.264 | 8.1186 | 0.0495 | 0.9999 |
| ENSMUSG00000054814 | *Usp46* | -0.251 | 6.5243 | 0.0495 | 0.9999 |
| ENSMUSG00000064380 | *Gm26448* | 0.594 | 2.4115 | 0.0495 | 0.9999 |
| ENSMUSG00000048701 | *Ccdc6* | 0.273 | 7.2865 | 0.0498 | 0.9999 |
| ENSMUSG00000031845 | *Bco1* | 0.757 | 1.3026 | 0.0499 | 0.9999 |
| ENSMUSG00000023452 | *Pisd* | 0.280 | 6.4225 | 0.0500 | 0.9999 |

Transcripts are sorted according to FDR values. ^a^ log2 Fold Change; ^b^ log2 Counts Per Million; ^c^ False Discovery Rate.
